# Patient-Perceived Physician Bias in Hidradenitis Suppurativa (HS)

**DOI:** 10.1007/s40615-024-02252-1

**Published:** 2024-12-09

**Authors:** Fatuma-Ayaan Rinderknecht, Haley Naik

**Affiliations:** 1 University of California San Francisco School of Medicine, San Francisco, CA USA; 2 Department of Dermatology, University of California San Francisco, 2340 Sutter Street, S429, San Francisco, CA 94143-0808 USA

**Keywords:** Public health, Diversity, Equity and inclusion, Implicit bias, Skin of color, Dermatology, Hidradenitis suppurativa

## Abstract

Hidradenitis suppurativa (HS) is a debilitating and understudied inflammatory skin disease that disproportionately impacts Black Americans. The objective of this study was to explore the role that race and ethnicity may play in HS patients’ perceptions of physician bias and their care quality. We administered a cross-sectional anonymous online survey to individuals with HS from June 13 to 30, 2021. Items from the Commonwealth Fund 2001 Health Care Quality Survey were employed to evaluate whether participants felt they were judged based on their race or ethnicity. Data was analyzed utilizing the test of equal or given proportions for assessing statistical significance with a threshold *p*-value < 0.05. The survey received a total of 1040 responses. The cohort was majority female and racially and ethnically diverse, with participants from six continents. Overall, 15.2% (136/894) of respondents reported feeling that they would receive better care if they were of a different race/ethnicity, and 13.6% (122/894) felt their primary HS provider treated them unfairly based on their race. Participants who belonged to minority groups more often reported feeling that they would receive better medical care if they were of a different race/ethnicity and felt that they were treated unfairly due to their race/ethnicity compared to White participants. These findings highlight the need to better understand the complex systemic and interpersonal factors at play in interactions between HS patients and healthcare providers to ensure that patients can receive much-needed care.

## Introduction

Hidradenitis suppurativa (HS) is a debilitating and understudied inflammatory skin disease that causes painful nodules and tunnels in the axillary, inguinal, and anogenital regions of the body. HS can have a profound negative effect on patients’ quality of life, often causing chronic pain, disability, and psychosocial challenges [1]. Previous studies of HS patients have largely focused on individuals with healthcare access, potentially overlooking those who face significant barriers to care [1]. Many patients with HS may be unable to access care due to financial constraints or distrust of the healthcare system [2, 3]. To address this gap, we utilized a Facebook survey to reach a large and diverse HS population, including those who may not typically engage with traditional healthcare settings.

HS disproportionately impacts Black Americans [4]. Black/African Americans with HS are also more likely to have more severe disease, in part due to lower socioeconomic status and poor access to care [5]. The objective of this study was to explore the role that race and ethnicity may play in HS patients’ perceptions of physician bias and care quality, broadening our understanding of how these factors influence access and treatment experiences.

## Methods

We administered a cross-sectional anonymous online survey to individuals with HS from June 13 to 30, 2021. The survey consisted of 35 items that collected participant demographics, HS disease characteristics, treatments received, sources of stigma, barriers to accessing HS care, and the type of primary provider managing their HS. Participant groups were identified using the keyword “hidradenitis suppurativa” in Facebook’s inbuilt search engine [6]. Group administrators of the participating online support groups were asked to share the survey link three times throughout the survey period. After the survey closed, the administrators provided post reach metrics, which is a Facebook application measure indicating how many individuals viewed the post at least once. Post reach data was utilized to estimate the response rate [6].

HS diagnosis was self-reported and confirmed either by validated screening questions or by participant report of medical provider diagnosis. Informed consent was obtained from all individual participants included in the study. Items from the Commonwealth Fund 2001 Health Care Quality Survey [7] were employed to evaluate whether participants felt they were judged based on their race or ethnicity [8].

Data was collected and stored in Qualtrics (Qualtrics, Provo, UT, USA). Responses of minority racial groups (Black, Hispanic/Latino, Asian, American Indian/Native Hawaiian/Pacific Islander) were compared against those of White patients to evaluate potential disparities utilizing the test of equal or given proportions for assessing statistical significance with a threshold *p*-value < 0.05 (RStudio, PBC, Boston, MA). The survey was determined to be exempt by the University of California, San Francisco Institutional Review Board (IRB #19–27407).

## Results

The estimated reach of the survey was 5168 views, and a total of 1040 responses were collected, yielding a response rate of 20.1%. The cohort was majority female (963/994, 97%) and racially and ethnically diverse (White 77% (767/996), Black 10.4% (104/996), Hispanic/Latino 7.2% (72/996), Asian 4.2% (42/996), American Indian/Alaskan Native 3.5% (37/996)). The majority of participants resided in North America (72%, 679/943) and Europe (22%, 205/943). Only 46.9% (445/949) had a dermatologist as their primary HS provider, while 15.2% (144/949) had no provider for their HS (Table 1).

**Table 1:** Cohort demographics

	Number	Percentage
Sex		
Female	963	96.7%
Male	31	3.2%
Race/ethnicity		
White	767	77.0%
Black	104	10.4%
Hispanic/Latino	72	7.2%
Asian	42	4.2%
American Indian/Alaskan Native	35	3.5%
Native Hawaiian/Pacific Islander	2	0.2%
Others/unknown/declined	51	5.1%
Geography		
Suburban	366	38.7%
Urban	308	32.6%
Rural	271	28.7%
Country		
North American	679	72.0%
Europe	205	21.7%
Asia	12	1.3%
Africa	10	1.1%
Central/South America/Caribbean	6	0.6%
New Zealand/Australia	25	2.7%
Other	6	0.6%
Primary HS provider type		
Dermatologist	445	46.9%
Non-dermatologist	360	37.9%
No provider	144	15.2%

Overall, 15.2% (136/894) of respondents reported feeling that they would receive better care if they were of a different race/ethnicity, and 13.6% (122/894) felt their primary HS provider treated them unfairly based on their race. Compared to White respondents, Black (White 7.8% vs Black 40.2%, *p* < 0.001), Hispanic/Latino (White 7.8% vs Hispanic/Latino 34.9%, *p* < 0.001), and American Indian/Native Hawaiian/Other Pacific Islanders (White 7.8% vs American Indian/Native Hawaiian/Other Pacific Islanders 32.4%, *p* < 0.001) respondents more often reported feeling that their primary HS provider treated them unfairly based on their race. However, there was no significant difference between White and Asian participants when asked if they felt that their HS provider treated them unfairly based on race (White 7.8% vs Asian 16.7%, *p* = 0.09). Compared to White respondents, Black (White 6.0% vs Black 63.0%, *p* < 0.001), Hispanic/Latino (6.0% vs 45.5%, *p* < 0.001), American Indian/Native Hawaiian/Other Pacific Islanders (6.0% vs 29.4%, *p* < 0.001), and Asian (6.0% vs 26.7%, *p* < 0.001) respondents more often reported that they believed they would receive better care if they were of a different race/ethnicity (Table 2).

**Table 2:** Patient-perceived physician bias. Proportions of survey respondents stratified by race who reported physician bias in the context of HS care. Proportions of each group were compared against those of White participants to assign a *p*-value

	Would receive better medical care if different race/ethnicity		*p*-value	Felt primary HS provider/staff judged you unfairly/treated you with disrespect based on race/ethnicity		*p*-value
Race	Yes	Freq (%)		Yes	Freq (%)	
Overall cohort	136/894	15.2%	0.06	122/894	13.7%	0.26
White	42/696	6.0%	-	54/696	7.8%	-
Black	58/92	63.0%	< 0.001	37/92	40.2%	< 0.001
Hispanic/Latino	30/66	45.5%	< 0.001	23/66	34.9%	< 0.001
Asian	8/30	26.7%	< 0.001	5/30	16.7%	0.09
American Indian/Native Hawaiian/Other Pacific Islanders	10/34	29.4%	< 0.001	11/34	32.4%	< 0.001
Others	7/39	18.0%	0.01	10/39	25.6%	0.001

## Discussion

This study surveyed a global cohort of racially and ethnically diverse people with HS from six continents about their perceptions of racial and ethnic bias in their HS care. Participants who belonged to minority groups more often reported feeling that they would receive better medical care if they were of a different race/ethnicity and felt that they were treated unfairly due to their race/ethnicity compared to White participants. There has been limited previous research on perceived physician bias among patients with dermatologic conditions. To our knowledge, this is the first study of this kind in a global HS cohort.

This study suggests that race may play a role in patients’ perceptions of their care. As HS disproportionately impacts historically marginalized groups, this is particularly important to consider in the care of people with HS. A 2017 study found that the overall prevalence of HS was 0.10%; however, this prevalence was higher among women (0.17%) and highest among Black people (0.30%) [4]. While there is limited data exploring perceived physician bias among dermatology patients, increased perceived physician bias among Black, Asian, and Latino patients compared to White patients has been documented in other specialties, which is consistent with our findings [8, 9]. A single study exploring perceived bias among dermatology patients found that among their cohort of 19 Black patients, patients were more likely to perceive that skin of color clinic (SOCC) dermatologists were better trained to care for Black patients and showed patients greater respect and dignity, compared to non-SOCC dermatologists [10]. This is particularly relevant in dermatology as dermatologic education and textbooks have historically focused on lighter skin tones, limiting exposure to the appearance of skin pathology across diverse and darker skin tones where disease may present differently [9]. This gap in training may lead to disparities in patient outcomes due to delayed diagnosis. SOCCs have emerged to ensure that trainees receive training in recognizing dermatologic disease in darker skin tones [11].

While reasons that minority patients in our cohort were more likely to perceive physician bias are unknown and likely multifaceted, some reasons may include historical mistreatment by physicians and implicit bias. Many minority groups have faced centuries-long historical mistreatment by the medical community, including but not limited to the Tuskegee study, where African American men with syphilis were denied treatment to study the progression of the disease, the forced sterilization of Black and Indigenous women, and medical experimentation on minorities without consent [12]. These historical injustices created a sense of distrust of medicine among many in minority communities and may add to present-day perceived physician bias. Previous research has also found that some physicians may practice with bias against minority patients. A study assessing implicit bias of internal and emergency medicine residents presented with a clinical vignette followed by a questionnaire and found that residents had an implicit preference favoring White Americans and implicit stereotypes of Black Americans as less cooperative [13]. A more recent study assessing the effects of patient skin tone on decision-making found that dermatologists were more likely to order labs for lighter-skinned patients with psoriasis and more likely to order HIV and RPR tests for teenagers with atopic dermatitis who had darker skin tones [14].

Notably, we found no significant difference in the perception of unfair treatment based on race/ethnicity between Asian and White patients. This may be due to higher rates of racial concordance among Asian patients and their doctors (69% racial concordance), compared to Black patients (22%) and Hispanic/Latino patients (40%), though rates are still lower than White patients (84% racial concordance) [15]. Racial concordance has been associated with stronger doctor-patient relationships and higher levels of patient satisfaction [15, 16].

This survey was limited by female predominance, self-selection bias, and cross-sectional study design. While utilizing Facebook as a platform for this study presents certain limitations, such as the difficulty in determining response rates, it also allows for a broader and more diverse reach of the survey population. Lastly, while this was a global study, the majority of patients were based in the USA and Europe and thus may limit the generalizability of our findings to other regions. Notably, racial and ethnic diversity within these regions, in addition to the disproportionately low number of Hispanic/Latino and Black physicians in the USA, may contribute to race-discordant doctor-patient relationships in these regions and possibly shape these interactions [17].

## Conclusion

This survey study of a diverse global cohort of HS patients illuminates the prevalence of perceived bias among racial and ethnic groups with HS. These findings highlight the need to better understand the complex systemic and interpersonal factors at play in interactions between HS patients and healthcare providers to ensure that patients can receive much-needed care. These findings underscore the importance of incorporating cultural humility into medical training, as well as investigating the complexities of perceived bias across diverse patient populations to advance health equity.

## Data Availability

Data can be available upon request.

## Code Availability

Data can be available upon request.
